# Modulation of antioxidant defense and PSII components by exogenously applied acetate mitigates salinity stress in *Avena sativa*

**DOI:** 10.1038/s41598-024-51302-5

**Published:** 2024-01-05

**Authors:** Sajeesh Kappachery, Mohamed AlHosani, Tanveer Alam Khan, Sara Nouh AlKharoossi, Nemah AlMansoori, Sara Ali Saeed AlShehhi, Hamda AlMansoori, Maha AlKarbi, Shina Sasi, Sameera Karumannil, Sampath Kumar Elangovan, Iltaf Shah, Mayank Anand Gururani

**Affiliations:** 1 Department of Biology, College of Science, United Arab Emirates University, P.O.Box 15551, Al Ain, UAE; 2 Department of Chemistry, College of Science, United Arab Emirates University, P.O.Box 15551, Al Ain, UAE; 3 Khalifa Center for Genetic Engineering and Biotechnology, College of Science, United Arab Emirates University, P.O.Box 15551, Al Ain, UAE

**Keywords:** Plant sciences, Photosynthesis, Plant physiology, Plant stress responses

## Abstract

Salinity stress has detrimental effects on various aspects of plant development. However, our understanding of strategies to mitigate these effects in crop plants remains limited. Recent research has shed light on the potential of sodium acetate as a mitigating component against salinity stress in several plant species. Here, we show the role of acetate sodium in counteracting the adverse effects on oat (*Avena sativa*) plants subjected to NaCl-induced salinity stress, including its impact on plant morphology, photosynthetic parameters, and gene expression related to photosynthesis and antioxidant capacity, ultimately leading to osmoprotection. The five-week experiment involved subjecting oat plants to four different conditions: water, salt (NaCl), sodium acetate, and a combination of salt and sodium acetate. The presence of NaCl significantly inhibited plant growth and root elongation, disrupted chlorophylls and carotenoids content, impaired chlorophyll fluorescence, and down-regulated genes associated with the plant antioxidant defense system. Furthermore, our findings reveal that when stressed plants were treated with sodium acetate, it partially reversed these adverse effects across all analyzed parameters. This reversal was particularly evident in the increased content of proline, thereby ensuring osmoprotection for oat plants, even under stressful conditions. These results provide compelling evidence regarding the positive impact of sodium acetate on various plant development parameters, with a particular focus on the enhancement of photosynthetic activity.

## Introduction

Abiotic stress encompasses a spectrum of non-living environmental factors that adversely affect plant growth. These stressors include extreme temperature, drought, heavy metals, and salinity. While each stress type elicits unique responses, there exists a commonality in the molecular mechanisms triggered by plants to cope with these challenges. Understanding the intricate signaling networks and molecular responses is fundamental to devising strategies for enhancing plant resilience. Among various abiotic stresses, salinity emerges as a major factor significantly impacting crop productivity and growth, with diverse harmful effects on plant development^1^. Estimates indicate that around 50% of agriculturally utilized land will likely be impacted by salinity by 2050^2^. Salinity, a primary contributor to the degradation of agricultural land, makes it unsuitable for crop cultivation and production^3^. Salinity presents various challenges, including disruptions to vital physiological functions in plants such as premature leaf senescence, membrane disruption, sodium ion toxicity, nutritional abnormalities, and leaf necrosis, ultimately leading to tissue death^4,5^. Additionally, heightened soil salt levels disturb root growth patterns, hinder water uptake, upset ion balance in plants, and cause oxidative damage, resulting in diminished yields of inferior quality^6,7^. Saline stress significantly impairs the growth of diverse crops, including strawberry, pea, potato, eggplant, and radish^8–11^. Despite these obstacles, plants can employ various mechanisms to withstand saline stress, a complex trait involving biochemical and physiological processes and the activation of multiple genes^12^. Elevated salinity in the soil disrupts water and nutrient uptake, resulting in the accumulation of harmful ion levels^13^. Maintaining the proper homeostasis of cellular ions is crucial for plant cell growth under salinity stress, necessitating a coordinated mechanism that includes vacuole compartmentalization, controlled ion uptake, and active ion extrusion into the external environment^14^. Addressing the urgent need to develop sustainable agricultural practices that can alleviate the adverse effects of salinity stress on crop production is essential. This becomes particularly crucial in meeting the rising global food demands and overcoming challenges posed by a growing population^13^.

Subsequently, the search for innovative and sustainable solutions has prompted researchers to delve into unconventional avenues, and one such avenue gaining attention is the role of sodium acetate (SA) in alleviating the adverse effects of abiotic stress on plants. Research suggests that SA may act as a modulator of plant responses to abiotic stress, influencing key physiological processes such as osmoregulation, ion homeostasis, and antioxidant defense mechanisms^15^. The intricate interplay between SA and plant metabolism under stress conditions forms the crux of ongoing investigations. To comprehend the impact of SA on plant resilience, researchers are exploring the molecular and biochemical pathways involved. Initial findings indicate that SA may enhance the synthesis of stress-responsive proteins, regulate the expression of genes associated with stress tolerance, and modulate ion transport across cellular membranes^15^. Unravelling these mechanisms is crucial for developing targeted strategies to optimize the use of SA in agriculture. Recently, there has been extensive documentation regarding the positive effects of externally applying acetate to alleviate the detrimental effects of drought stress in *Arabidopsis thaliana*, *Manihot esculenta*, and *Glycine max*^16–18^. This treatment has also demonstrated efficacy in mitigating salinity and copper stress in *Lens culinaris* plants^19,20^, addressing salinity stress in *Vigna radiata* and *Lolium perenne*^21,22^, and countering cadmium stress in rice^23^. These findings emphasize the versatility and potential of applying acetate to manage various abiotic stresses in crop cultivation. The capacity of acetate-induced abiotic stress tolerance has been observed across a diverse range of plant species, exhibiting a positive correlation with increased root and shoot biomass, elevated levels of photosynthetic pigments, enhanced leaf relative water content, and improved hormonal metabolism, while simultaneously reducing oxidative damage^18,22^. Similarly, the upregulation of genes associated with acetic acid biosynthesis significantly enhances drought tolerance, as evidenced in a study involving *Arabidopsis* transgenic plants expressing *PYRUVATE DECARBOXYLASE* (PDC1) or *ALDEHYDE DEHYDROGENASE 2B7* (ALDH2B7) under the control of the drought-inducible *TRYPTOPHAN-RICH SENSORY PROTEIN* (TSPO) promoter^15^. The authors suggest that the increased acetic acid content in plant cells may function as an energy source, ensuring sufficient cellular energy during stressful conditions^15^.

Oats hold a pivotal role as a cereal crop for their high nutritional content and positive impacts on human health and livestock productivity^24^. However, unsustainable irrigation practices, the presence of hazardous sodium-containing salts in irrigation water, and rising water tables are expected to intensify the issue of saline soils in the coming years^25^. The potential of acetate in mitigating salinity stress in oats remains unexplored in the existing literature. Consequently, our study aims to investigate the potential of acetate as a saline stress alleviator in oats, to propose an economically viable and efficient solution to managing excessive salinity. Our findings present the first reported evidence of the advantageous effects of acetate in mitigating the damage induced by salinity stress in oat plants.

## Materials and methods

### Plant materials and growth conditions

Oat seeds were acquired from a local agricultural market in Al Ain, United Arab Emirates. The study was carried out with a completely randomized design and three replications, applying a 2 × 4 factorial experiment. The seeds were sown in pots with a diameter of 25 cm filled with sterile soil peat (Van Egmond, Potgrond, Netherlands), and germination was observed within a period of 3 to 4 days. The trial was conducted under greenhouse conditions (day/night/ 25 °C/16 °C, 16/8 h light/dark, 70% air relative humidity and 300 µmol m^−2^ s^−1^ of light intensity). Three weeks-old plants with uniform size were used for the experiments. In our current study, we selected the most effective combination of sodium acetate (SA) concentration, determined to be 10 mM through preliminary investigation involving various concentrations. The experimental design included four sets of plants, each with triplicate samples. The first set was exclusively irrigated with tap water (WW), while the second set (NaCl) got tap water infused with gradually rising NaCl concentrations of 50 to 400 mM with an increment of 50 mM every alternative day of irrigation, once the progressive increment of salt concentration in the irrigation water reaches 400 mM (by two weeks), the plants were continued to irrigate with the same concentration of salt for the rest of the experiment.

The third set (NaCl + SA) was subjected to 10 mM sodium acetate mixed with tap water containing varying NaCl concentrations progressively increasing as stated above. In the fourth set, sodium acetate (SA) was directly applied to tap water, which served as an irrigation medium. After three weeks of treatment, the samples were collected and subjected to physiological and biochemical evaluations. The experiment were continued for two more weeks and photographs were taken to record the overall impact of different treatments on growth of oat plants. Experiment were repeated three times to confirm the findings.

### Measurement of growth parameters

After 21 days of stress treatment, the shoot length, plant fresh weight (FW), and plant dry weight (DW) were determined. The shoots and roots were rinsed with distilled water and then placed in paper towels to eliminate any surface moisture before measuring the FW of the plants using an electric balance in three replications. The samples were subsequently dried for 48 h at 80 °C to obtain the DW measurement.

### Estimation of chlorophylls and carotenoids

Chlorophylls and carotenoids extraction were carried out from 100 mg of leaf samples grounded in 2 mL of 80% acetone and further centrifuged at 3000 rpm for 15 min. The supernatant was collected and the procedure was repeated until the green color was removed from the tissue. All the extracts’ supernatants were pooled and used for pigment quantification. Spectrophotometer measurements were performed at the absorbance of 663, 645 and 470 nm. Pigment contents were determined as described earlier^26^.

### Estimation of ion leakage, stomatal conductance, MDA and Proline contents

Electrolytic leakage was measured by suspending leaf discs in 10 mL of distilled water in a test tube using the formula, Electrolyte leakage (%) = (ECb-ECa/ECc) X 100. Where ECa is electric conductivity of the water measured after 30 min of suspending the leaf discs at room temperature, ECb is electric conductivity after 30 min incubation at 55 °C, and (ECc) is electric conductivity after 10 min incubation at 100 °C.

Stomatal conductance on the upper surface of the fully expanded leaves was measured using a steady-state diffusion leaf porometer (SC-1; Decagon Devices, USA). Before each day´s data collection, the porometer was calibrated. All measurements were conducted at a temperature of 25 ± 1 °C and a relative humidity of 55 ± 5 °C. For determination of MDA equivalent content, 500 mg of leaf tissue was ground to a fine powder using liquid nitrogen and subsequently homogenized in 5 mL of a 50 mM buffer (0.07% NaH_2_PO_4_·2H_2_O and 1.6% Na_2_HPO_4_·12H_2_O). The homogenized samples were centrifuged at 13,000 rpm for 25 min at 4 °C and supernatant was collected. 1 mL of the supernatant was mixed with 4 mL of a solution containing 20% trichloroacetic acid and 0.5% thiobarbituric acid. After incubating the reaction mixture at 95 °C for 30 min, it was promptly placed on ice for 10 min. The reaction mixture was then centrifuged for 10 min at 13,000 rpm, and the absorbance readings at 532 and 600 nm were recorded. The absorbance measurement at 532 nm was subtracted from the non-specific absorption at 600 nm. The final MDA concentration of the samples was calculated as previously described^27^.

Proline estimation in leaf samples collected from WW, SA, NaCl and NaCl + SA treatments was conducted via a colorimetric method. To extract proline, 0.5 g of leaf tissue was ground and homogenized in 10 mL of 3% aqueous sulfosalicylic acid, followed by filtration through Whatman No.1 filter paper. Equal volumes of the filtrate and acid ninhydrin reagent prepared in glacial acetic acid and phosphoric acid were mixed and incubated for 1 h. Following the incubation period, 4 mL of toluene was introduced to terminate the reaction. The toluene layer turned blue violet indicating the presence of proline. The absorbance of chromophore-containing extract was spectrophotometer measured at 520 nm. To determine the proline concentration in μmol g − 1 FW (fresh weight), a standard curve was constructed using known proline concentrations and their corresponding absorbance values^28^.

### Estimation of phytohormones

The analysis of the plant hormones namely Indol-3 acetic acid (IAA), and abscisic acid (ABA) was conducted by an In-house fully validated Liquid Chromatography-Mass Spectrometry/Mass Spectrometry (LC-MSMS) method. The leaves of oat plants were flash-frozen and subsequently ground into powder using liquid nitrogen. For the extraction, 1 g of lyophilized sample was employed along with additional internal standards for IAA (Indoleacetic Acid-d4) and ABA (Abscisic acid-d6). A mixture comprising 5.0 mL of 1% acetic acid in acetonitrile was added sample and vigorously vortexed. The mixture was centrifuged at 1500xg for 10 min at 4 °C. After centrifugation, the supernatant was collected and mixed with 1 mL of mobile phase. 5 µL of the prepared was injected into the LC-MS-MS encompassed an ultra-pressure liquid chromatography (UPLC) system, specifically the model LC-30AD (Nexera X2) binary pump from Shimadzu (Japan), coupled with a tandem mass spectrometer 8060 (Shimadzu, Japan). The mass spectrometer operated in positive electrospray ionization (ESI) mode, with nitrogen serving as the heating and drying gas.

A chromatographic separation was accomplished using a reverse phase column, Acquity UPLC®BEH C18 (2.1 * 50 mm, 1.7 µm). The column was held at 30 °C and the mobile phase used in the chromatography consisted of a mixture of 0.01% Formic Acid in water and Methanol (35:65) respectively. The quantification and separation of phytohormones were achieved through multiple reaction monitoring modes (MRM) of their [M + H] + ions. Each sample was analysed in triplicate.

### Expression analysis of genes encoding ROS-scavenging enzymes

The total RNA of both control and treated samples was extracted using the Plant RNA Extraction Kit (NorgenBiotek, Canada). The quantity and quality of the extracted RNA were checked by Nanodrop 2000 and gel electrophoresis. cDNA was synthesized from 1 μg total RNA using the TruScript First Strand cDNA Synthesis Kit (NorgenBiotek, Canada) following the manufacturer’s instructions. The qRT-PCR reactions were carried out in a 20 μL volume containing 2 μL of diluted cDNA (10X), 200 nM of primers and SYBR™ Green PCR Master Mix (Applied Biosystem, USA) in an optical 96 well plate using StepOnePlus™ Real-Time PCR System (Applied Biosystem, USA). The primers used in the reactions are listed in Table S1. RT-PCR temperature cycle was set up as one cycle at 95 °C for 3 min, 40 cycles of 94 °C for 30 s, 58 °C for 15 s, and 72 °C for 20 s. The transcript level of the target gene was compared by the 2^−ΔΔCt^ method^29^. *GLYCERALDEHYDE-3-PHOSPHATE DEHYDROGENASE* (*GAPDH*; NCBI Acc. No. MH260251) was used as an endogenous reference gene for normalization and calculating the relative fold change of gene of interest. Each biological sample was analyzed in three technical replicates.

### Chl-a fluorescence kinetics

To ensure the closure of all PSII (Photosystem II) reaction centres, plants subjected to salinity stress were placed in darkness for 1 h. Chlorophyll-a (Chl-a) fluorescence measurements were conducted using a Pocket PEA (Plant Efficiency Analyzer, Hansatech, UK), adhering to the manufacturer’s guidelines. The fluorescence of experimental plants was acquired at a wavelength of 685 nm using an actinic light of 3000 mol photons m^−2^ s^−1^. Fluorescence data was averaged from five measurements per plant in triplicate. Maximal fluorescence (F_m_) and minimal fluorescence (F_0_) were utilized to calculate the quantum yield of photochemistry (F_v_/F_m_) ratio. The data analysis was carried out using the BIOLYZER program and the JIP-test equation denoted φP_x_ = TR_x_/ABS = 1 − F_x_/F_m_^30^. The actual quantum yield of primary photochemistry is derived using the theory of energy fluxes on bio membranes^31^. The theory of energy fluxes on biological membranes underpins the derivation of the actual quantum yield of primary photochemistry, revealing how energy fluxes from photon absorption (ABS) and electron transport (ET) combine to yield free energy flux (RE) once the electron acceptor of photosystem I (PSI) is reduced. The specifics of how energy is transferred from PSII reaction centres (RCs) to photosynthetic electron transport chains were also revealed by BIOLYZER, in terms of several crucial photosynthetic parameters.

### Statistical analyses

The data obtained from all the experiments carried out for this study were analyzed using GraphPad Prism 9.0 after being repeated at least three times. Tukey's multiple comparison tests were used after the one-way analysis of variance to determine statistical differences. For each experiment, the standard error was determined using the *n* values. Significant differences at *P* < 0.05 were calculated and indicated by bars in the figures with different letters.

### Ethics approval

All the methods were carried out in accordance with relevant institutional guidelines and regulations.

## Results

### Application of sodium acetate enhances growth parameters in oat plants under salt stress conditions

**Figure 1:**
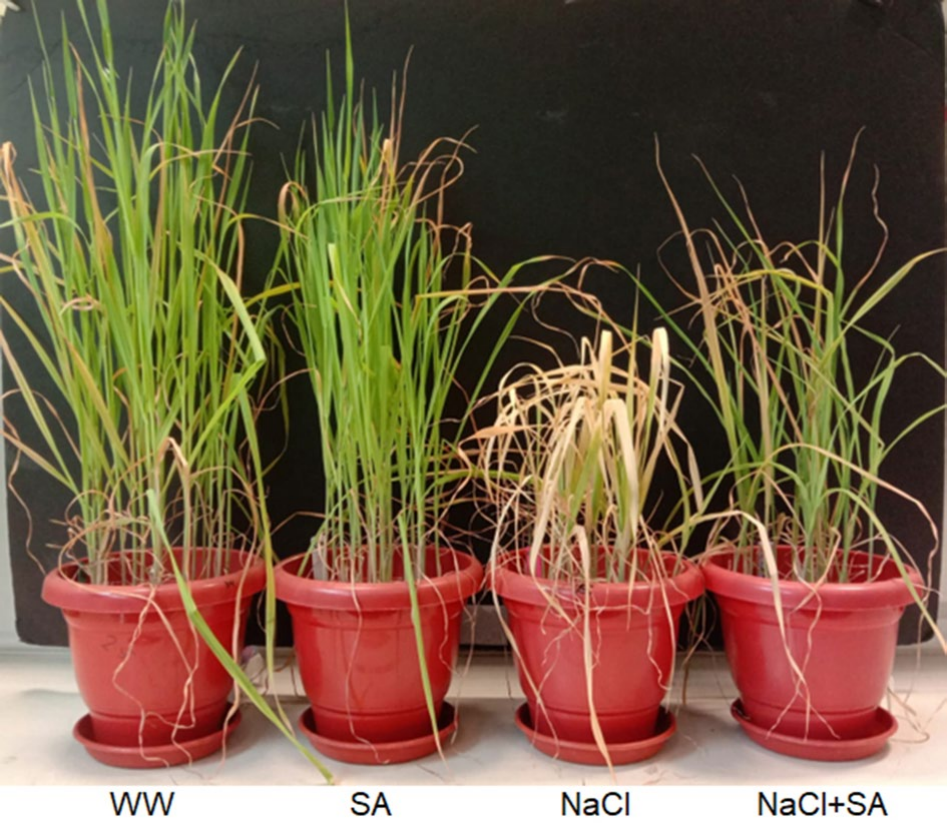
The Impact of Different Treatment on Growth and Morphology of Oat Plants. WW, well-watered oat plants (control); NaCl, oat plants subjected to salinity stress for 5 weeks; NaCl + SA, oat plants irrigated with 10 mM sodium acetate (SA) mixed with tap water containing NaCl for 5 weeks, and SA, plants irrigated with 10 mM sodium acetate for 5 weeks.

**Figure 2:**
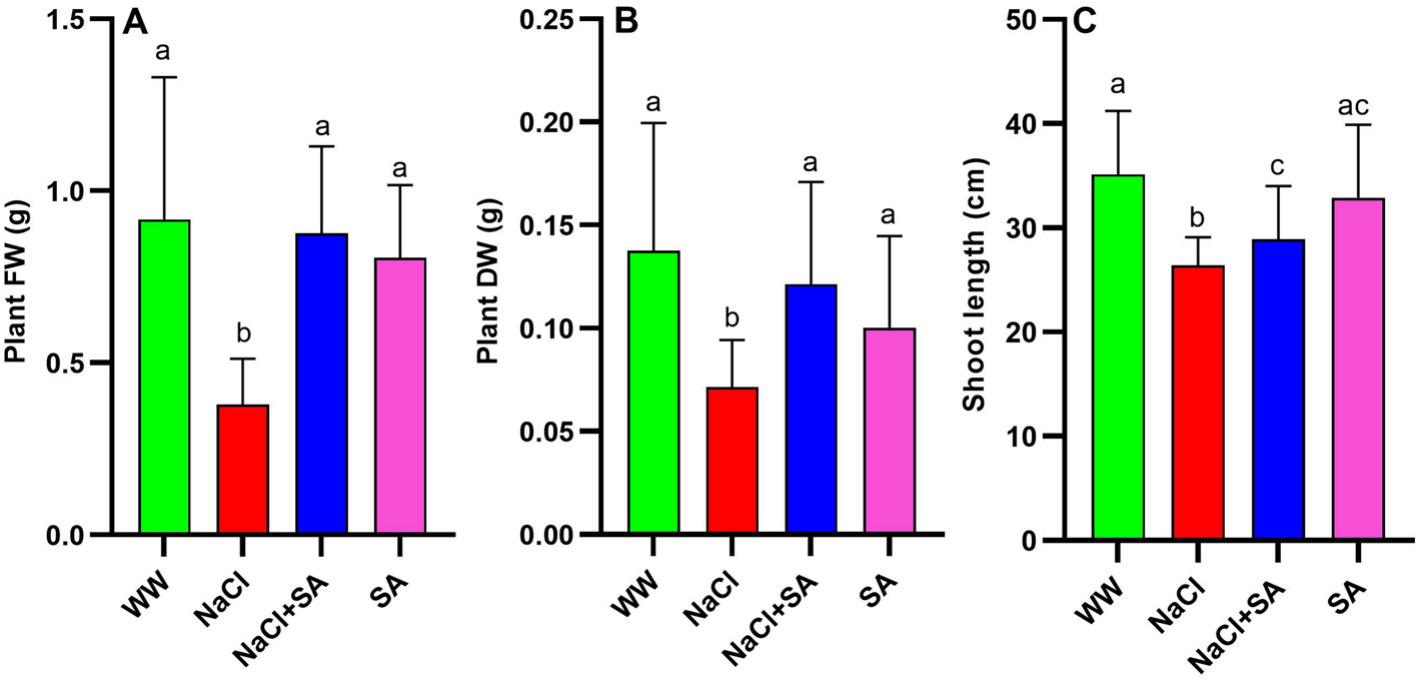
Impact of sodium acetate application on the growth parameters in oat plants subjected to salinity stress induced by NaCl. (**A**) Plant fresh weight (FW), (**B**) Plant dry weight (DW) and (**C**) Shoot length of oat plants. WW, well-watered oat plants (control); NaCl, oat plants subjected to incremental salinity stress, 50–400 mM NaCl, for 3 weeks; NaCl + SA, oat plants irrigated with 10 mM sodium acetate (SA) mixed with tap water containing varying NaCl concentrations, 50–400 mM, for 3 weeks, and SA, plants irrigated with 10 mM sodium acetate for 3 weeks. Values represent the average ± SE of three independent assays of four replicates in each treatment group. Different letters in each row indicate significant differences (*p* ≤ 0.05) between treatments; Tukey’s test (n = 8).

To explore the impact of sodium acetate application on mitigating the detrimental effects of salt stress in oat plants, three-week-old plants were subjected to a progressive salinity stress gradient ranging from 50 to 400 NaCl in the presence and absence of a 10 mM sodium acetate. Each plant group displayed distinct morphological traits The WW (Well-watered) and SA (Sodium acetate-treatment) groups exhibited a healthy appearance, whereas the NaCl (Sodium Chloride-treatment) plants showed signs of stress with yellowing of leaves and reduced size (Fig. 1). Remarkably, the growth of NaCl + SA (Sodium Chloride with Sodium Acetate-treatment) plants surpassed that of the NaCl-treated group with partial recovery from salt stress condition (Fig. 1). An enhancement in both fresh weight (FW) and dry weight (DW) was observed in WW-, SA- and NaCl + SA-treated plants when compared to NaCl counterparts (Fig. 2A,B). The average shoot length of WW and SA plants was significantly longer than that of NaCl- and NaCl + SA-treated plants (Fig. 2C). These results indicate the application of SA partial recovery to the harmful morphological traits triggered by salt stress.

### Sodium acetate treatment improves pigment accumulation, electrolyte leakage, proline accumulation, and MDA levels under salt stress conditions

**Figure 3:**
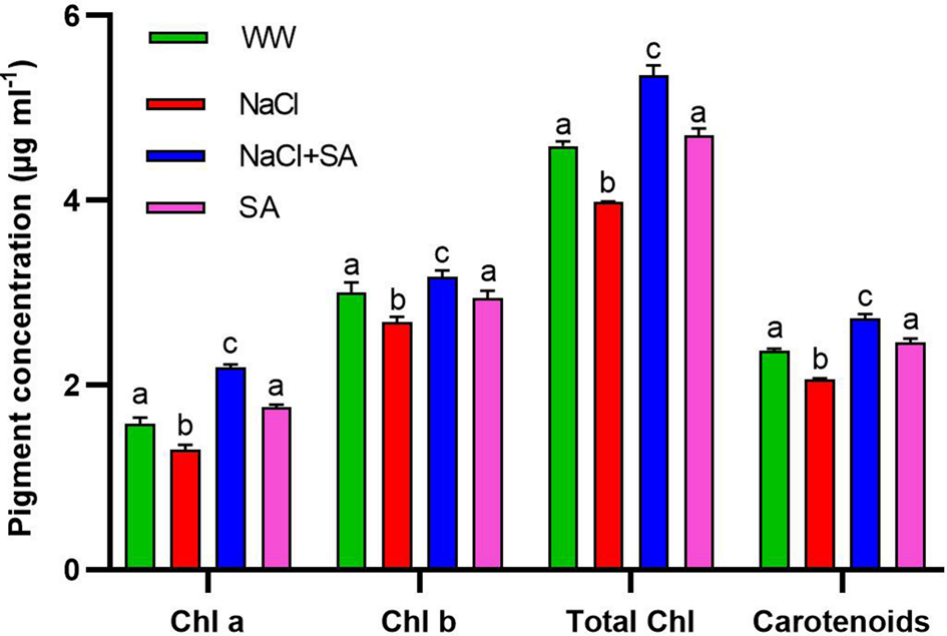
Effect of Sodium Acetate Application on Pigment Accumulation in Oat Plants Under Salinity Stress Induced by NaCl. Chla, chlorophyll a; Chlb, chlorophyll b; Total Chl, total chlorophyll. WW, well-watered oat plants (control); NaCl, oat plants subjected to incremental salinity stress, 50–400 mM NaCl, for 3 weeks; NaCl + SA, oat plants irrigated with 10 mM sodium acetate (SA) mixed with tap water containing varying NaCl concentrations, 50–400 mM, for 3 weeks, and SA, plants irrigated with 10 mM sodium acetate for 3 weeks. Values represent the average ± SE of three independent assays of four replicates in each treatment group. Different letters in each row indicate significant differences (*p* ≤ 0.05) between treatments; Tukey’s test (n = 3).

**Figure 4:**
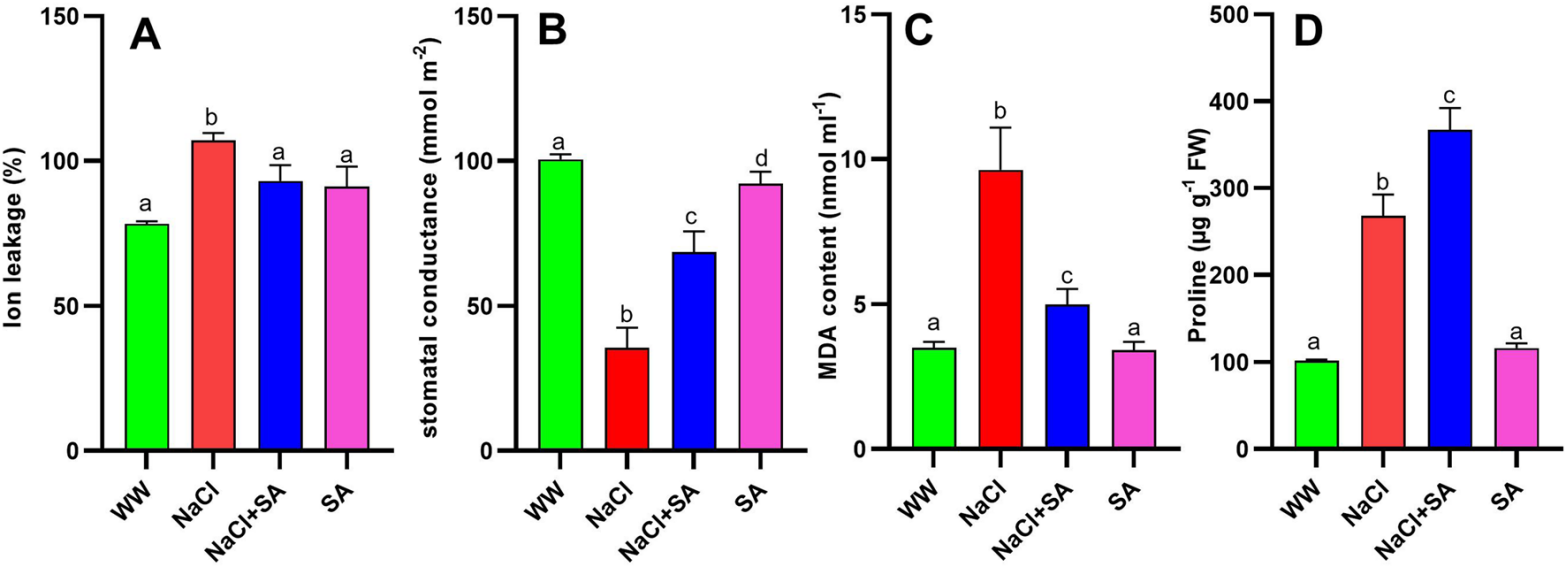
Effect of sodium acetate application on four physiological parameters across the four treatments groups in oat plants. (**A**) Ion leakage (**B**) stomatal conductance, (**C**) MDA content and (**D**) Proline accumulation. WW, well-watered oat plants (control); NaCl, oat plants subjected to incremental salinity stress, 50–400 mM NaCl, for 3 weeks; NaCl + SA, oat plants irrigated with 10 mM sodium acetate (SA) mixed with tap water containing varying NaCl concentrations, 50–400 mM, for 3 weeks, and SA, plants irrigated with 10 mM sodium acetate for 3 weeks. Values represent the average ± SE of three independent assays of four replicates in each treatment group. Different letters in each row indicate significant differences (*p* ≤ 0.05) between treatments; Tukey’s test (n = 3).

Pieces of evidence about the decline in photosynthetic parameters as an initial response to severe stress prompted our investigation into the effects of sodium acetate (SA) treatment on oat plants subjected to salt stress induced by NaCl on pigment accumulation and electrolyte leakage parameters. After three weeks of exposure to salt-induced stress, leaves of NaCl + SA-treated plants exhibited the highest chlorophyll content, whereas the NaCl-treated plants had the lowest. Remarkably, NaCl + SA-treated plants exhibited a 16.8% increase in chlorophyll accumulation compared to the WW-treated plants plants. Additionally, NaCl + SA-treated plants displayed a 14.8% higher accumulation of carotenoids when compared to the WW plants (Fig. 3). After three weeks, the impact of salinity stress on electrolyte leakage significantly increased when compared to their WW-treated counterparts. Interestingly, there was no notable difference in terms of electrolyte leakage between NaCl + SA and SA-treated plants (Fig. 4A). In WW-treated plants, the average stomatal conductance values were similar to those observed in SA plants. In addition, NaCl + SA plants exhibited a 35% reduction in stomatal conductance, whereas NaCl-treated plants showed a more substantial 60% reduction (Fig. 4B). To assess the impact of salinity stress on membrane lipid peroxidation, we measured the levels of MDA equivalents. Notably, the MDA equivalent values in the SA-treated plants closely resembled those of the WW-treated plants. However, a significant 48.1% reduction was observed in the NaCl + SA-treated plants compared to the NaCl-treated plants (Fig. 4C).

In order to gain insights into the biochemical mechanisms underlying the protective effects of SA treatment against physiological damage caused by salt stress, we assessed the levels of osmoregulatory proline in both treatment groups. The proline levels were quantified in both treated and untreated leaves, as shown in Fig. 4D. In contrast to the levels observed in WW- and SA-treated plants, the NaCl + SA-treated plants exhibited the highest concentration of proline followed by NaCl-treated plants (Fig. 4D). This heightened proline accumulation in NaCl + SA plants underscores its significance in enhancing plant stress tolerance.

### SA treatment improves the expression of antioxidant enzymes in plants under salt stress

**Figure 5:**
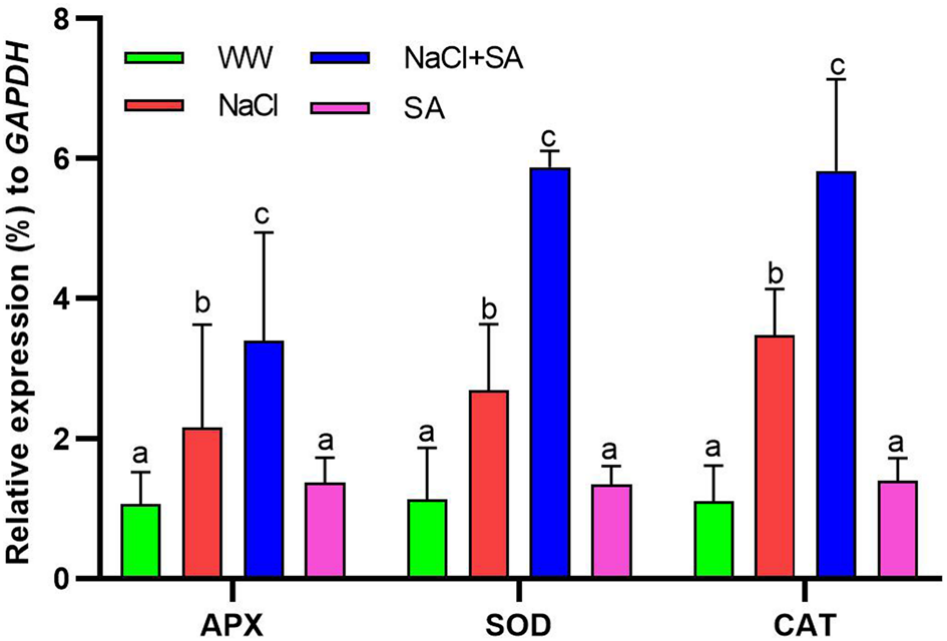
Relative mRNA level ROS-scavenging enzymes encoding genes in WW (well-watered oat plants, control), NaCl (oat plants subjected to incremental salinity stress, 50–400 mM NaCl), NaCl + SA (oat plants irrigated with 10 mM sodium acetate (SA) mixed with tap water containing varying NaCl concentrations, 50–400 mM, for 3 weeks) and SA (plants irrigated with 10 mM sodium acetate for 3 weeks). Fold expression values were normalized to those of the *GAPDH* control. Values represent the average ± SE of three independent assays of four replicates in each treatment group. Different letters in each row indicate significant differences (*p* ≤ 0.05) between treatments; Tukey’s test (n = 3). APX—ASCORBATE PEROXIDASE; SOD = SUPEROXIDE DISMUTASE; CAT = CATALASE.

After 3 weeks, an examination of gene expression involving the *ASCORBATE PEROXIDASE* (APX), *SUPEROXIDE DISMUTASE* (SOD), and *CATALASE* (CAT) enzyme-encoding genes revealed that there were no significant differences in the expression of these genes between the WW- and SA-treated plants (Fig. 5). However, remarkable differences were observed comparing the NaCl- and NaCl + SA-treated plants. In the case of NaCl + SA-treated plants, there was an upregulation of APX, SOD, and CAT, with levels being 1.6-, 2.2- and 1.6-fold higher, respectively, when compared to NaCl-treated plants (Fig. 5).

### SA application modulated IAA and ABA accumulation

**Figure 6:**
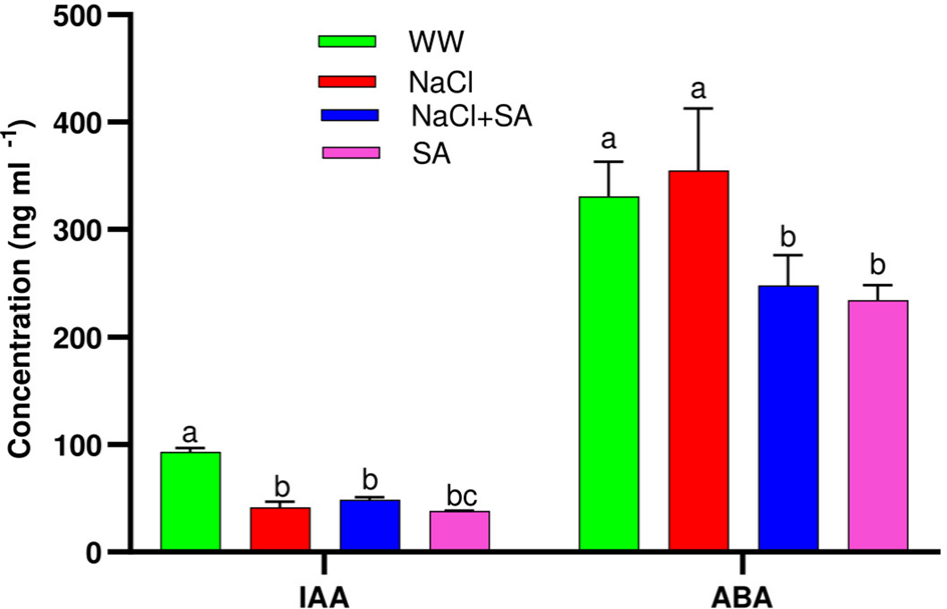
The Impact of Sodium Acetate Application on the Accumulation of Indole-3-Acetic Acid (IAA) and Abscisic Acid (ABA) in Oat Plants Across the Four Treatment Groups. WW, well-watered oat plants (control); NaCl, oat plants subjected to incremental salinity stress, 50–400 mM NaCl, for 3 weeks; NaCl + SA, oat plants irrigated with 10 mM sodium acetate (SA) mixed with tap water containing varying NaCl concentrations, 50–400 mM, for 3 weeks, and SA, plants irrigated with 10 mM sodium acetate for 3 weeks. Values represent the average ± SE of three independent assays of four replicates in each treatment group. Different letters in each row indicate significant differences (*p* ≤ 0.05) between treatments; Tukey’s test (n = 3).

Among plant hormones, auxins and abscisic acid play significant roles in response to salt stress in crop plants^32,33^. In this study, we investigated the levels of indole-3-acetic acid (IAA) and abscisic acid (ABA) in oat plants under salt stress conditions in the presence of sodium acetate. Our findings revealed a significant decline in IAA content across all treatment groups, NaCl, SA, and NaCl + SA, as compared to WW-treated plants (Fig. 6). Among these treatments, IAA's lowest accumulation was observed in NaCl-treated plants, while the highest was recorded under control conditions. Furthermore, we observed a positive correlation between auxin accumulation and growth rate, suggesting a role for auxin in root morphogenesis. Additionally, a reduction in ABA content in plants treated with SA and NaCl + SA when compared to WW-treated plants was observed, suggesting the regulatory influence of SA on ABA levels in the context of salt stress.

### SA improves the photosynthetic performance of plants under salt stress

To gain insights into whether sodium acetate treatment enhances photosynthesis in salt-stressed plants, we assessed the photosynthetic performance of both SA-treated and untreated plants exposed to salinity stress. Our evaluation involved measuring two key parameters: the photochemical efficiency of the photosystem (Fv/Fm) and the performance index (PI). The Fv/Fm is a crucial indicator of the photoinhibitory effects on the electron transport system resulting in various stress conditions reflecting the health and functionality of the photosystem in the plant system. On the other hand, the PI parameter predicts changes in fluorescence caused by variations in antenna conformation and energy fluctuations.

**Figure 7:**
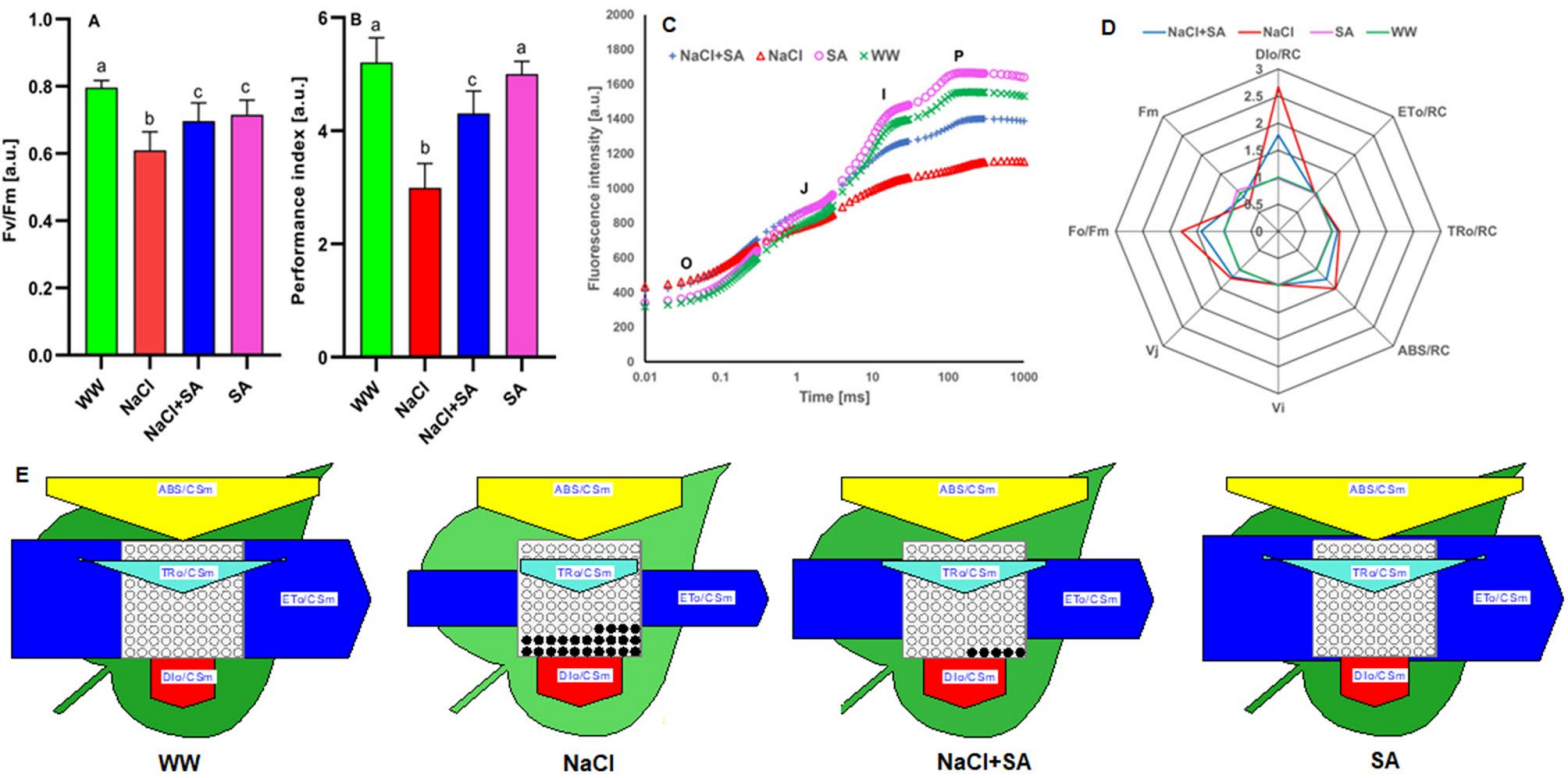
Fast chlorophyll A Fluorescence Kinetics (OJIP) in Dark-adapted Oat Leaves from the four experimental groups. (**A**) Quantum yield of PS II determined as Fv/Fm, (**B**) Performance index (PI), (**C**) The transient polyphasic curves for each line represent the average of 21 measurements, obtained from three replicates, each contaning seven plants with respective groups. (**D**) Biophysical parameters of PSII. (**E**) The energy pipeline leaf model of phenomenological fluxes (per cross-section, CSm) in four groups. The relative changes in arrow witdth depict the value of each parameter. Active Rcs are depicted as open circle, while inactive RCs are represented as closed circle. WW, well-watered oat plants (control); NaCl, oat plants subjected to incremental salinity stress, 50–400 mM NaCl, for 3 weeks; NaCl + SA, oat plants irrigated with 10 mM sodium acetate (SA) mixed with tap water containing varying NaCl concentrations, 50–400 mM, for 3 weeks, and SA, plants irrigated with 10 mM sodium acetate for 3 weeks. Values represent the average ± SE. Different letters indicate significant differences based on one-way ANOVA followed by Tukey's post hoc test at *p* < 0.001 (n = 21).

When plants were subjected to abiotic stress conditions, a significant shift in the Fv/Fm ratio, ranging from 0.58 to 0.7, was observed. Comparing the Fv/Fm ratios between WW-treated plants and NaCl-stressed ones, the application of SA had a minimum impact on the maximum photochemical efficiency. This trend was similarly reflected in the PI values of these plants (Fig. 7A,B). Notably, the PI values for NaCl + SA-treated plants were significantly higher compared to those of plants exposed solely to NaCl-induced salt stress.

Chlorophyll-a fluorescence transients of dark-adapted leaves for all plant groups are shown on a logarithmic scale from 20 μs to 1 s in Fig. 7C. These transient exhibits a typical OJIP shape, indicating the photosynthesis activity of all samples with similar maximum variable fluorescence (FM − F0 = FV). However, a notable distinct emerges when we focus on the transients between the J and P steps. Specifically, the NaCl-treated plants (red curve) demonstrated reduced photosynthetically active compared to the NaCl + SA-treated plants (blue curve) (Fig. 7C). Further analysis through JIP-assentaments of these plants groups of four groups under both normal and salt stress conditions highlights the significant enhancement in the absorption flux of photons per active RCs due to the application of SA during salt-stress conditions (Fig. 7D). In terms of specific parameters related to (ABS), trapping (TRo), electron transport (ETo) and the dissipation of excess energy (Dio) per RC, variations are observed between NaCl- and NaCl + SA-treated plants. ABS, TRo and DIo values were higher in the NaCl-treated plants, while Eto values remained consistent across all the plant groups (Fig. 7D). Regarding the parameters Vi and Vj, which correspond to the relative variations in fluorescence at the I and J-steps of OJIP transient, no significance differences are noted between NaCl- and NaCl + SA-treated plants (Fig. 7D). NaCl-treated plants exhibit a substantially higher Fo/Fm ratio, indicative of destabilization of photosynthetic apparatus^34^. This finding underscores the significant destabilization of the photosynthetic apparatus in NaCl-treated plants. Conversely, the maximum fluorescence (Fm) is notably lower in NaCl-treated plants compared to their NaCl + SA-treated counterparts (Fig. 7D), a trend also evident in the fluorescence transient curves presented in Fig. 7C.

Phenomenological energy fluxes, including ABS/CSm, TRo/CSm, ETo/CSm, and DIo/CSm were presented in Fig. 7E. These fluxes provide insights into the response of active reaction centres to light. Specifically, ABS/CSm signifies an increase in the number of active reaction centres, TRo/CSm indicates the trapped electron flux per reaction centre, and ETo/CSm represents the electron transport flux per reaction centre. Furthermore, DIo/CSm reflects the total energy dissipated per reaction centre. The varying widths of the arrows in the figure denote the magnitude of each parameter. A distinction between NaCl- and NaCl + SA-treated plants was observed (Fig. 7). ABS/CSm, TRo/CCSm, and ETo/CSm were significantly reduced in NaCl-treated plants compared to their NaCl + SA-treated counterparts. This suggests that the application of SA has a positive impact on the number of active reaction centres, the trapped electron flux per reaction centre, and the electron transport flux per reaction centre. Remarkably, all four plant groups exhibit similar DIo/RC values, indicating comparable levels of total energy dissipation per reaction centre. Furthermore, NaCl + SA-treated plants exhibit a substantial increase in the number of active reaction centres, as denoted by open circles, whereas NaCl-treated plants show an increase in the number of inactive reaction centres, indicated by filled circles. This finding underscores the beneficial effect of SA in promoting the activation of reaction centres under salt stress conditions (Fig. 7D).

Table 1 provides a comprehensive overview of the efficiency of key photosynthetic processes, assessed through ten critical parameters. The area parameter quantifies the total complementary area between the fluorescence induction curve and Fm. Additionally, the reduction in Fv/Fo, which signifies the ratio between the rate constants governing photochemical and nonphotochemical deactivation of excited chlorophyll molecules, exhibited a 55% decrease in NaCl + SA-treated plants, and a 143.4% increase in NaCl-treated plants. The fraction of active PSII RCs per effective antenna size is described as RC/ABS^35^ displayed a decrease of 122% in NaCl-treated plants and 22% less in NaCl + SA-treated plants compared to WW-treated plants. The parameter SFI(abs), representing a PSII structure–function-index^36^ represents a decline in plants under NaCl conditions. However, this decline was less pronounced at 12% in NaCl + SA-treated plants compared to a 36% reduction in NaCl-treated plants. Similar trends were observed in the ɸ(Po), ɸ(Eo), Ψo, and ɸ(Do) parameters, which delineate various aspects of the quantum yield of PSII^28,37^. Furthermore, the parameter Sm indicating the complementary area of the plastoquinone pool between PSII and PSI, exhibited an 82% increase in NaCl-treated plants and a 61% increase in NaCl + SA-treated plants, relative to the values observed in WW-treated plants.

**Table 1:** Impact of sodium acetate treatment on various chlorophyll-a transients in oat plants exposed to salinity stress conditions.

	Area	Fv/F0	RC/ABS	PI (ABS)	SFI (abs)	ɸ (Po)	Ψo	ɸ (Eo)	ɸ (Do)	Sm
WW	17,983.90 ± 326.48^a^	2.67 ± 0.06^a^	3.34 ± 0.06^a^	12.26 ± 0.74^a^	1.4 ± 0.04^a^	0.72 ± 0.004^a^	0.57 ± 0.003^a^	0.41 ± 0.004^a^	0.27 ± 0.004^a^	13.8 ± 0.11^a^
NaCl	12,435.47 ± 710.92^b^	0.44 ± 0.14^b^	0.81 ± 0.23^b^	7.56 ± 0.65^b^	0.2 ± 0.07^b^	0.2 ± 0.05^b^	0.33 ± 0.04^b^	0.09 ± 0.02^b^	0.8 ± 0.053^b^	33.06 ± 1.18^b^
NaCl + SA	15,960.66 ± 459.72^c^	1.51 ± 0.13^c^	2.59 ± 0.15^c^	8.84 ± 0.7^bc^	0.82 ± 0.08^c^	0.58 ± 0.02^c^	0.51 ± 0.006^ac^	0.3 ± 0.013^c^	0.42 ± 0.02^c^	26.31 ± 1.86^b^
SA	16,859.05 ± 488.32^ac^	2.69 ± 0.11^a^	3.28 ± 0.12^a^	12.16 ± 1.32^ac^	1.35 ± 0.08^a^	0.72 ± 0.007^a^	0.56 ± 0.007^ac^	0.4 ± 0.01^a^	0.27 ± 0.007^a^	13.26 ± 0.17^a^

Each experiment comprises three replicates, each consisting of seven plants. The presented values are expressed as the mean ± standard error (SE). Distinct letters denote statistically significant differences, as determined by one-way ANOVA followed by Tukey’s post hoc test (*p* < 0.001, n = 21).

### Sodium acetate modulates the expression of PSII proteins in plants under salt stress

**Figure 8:**
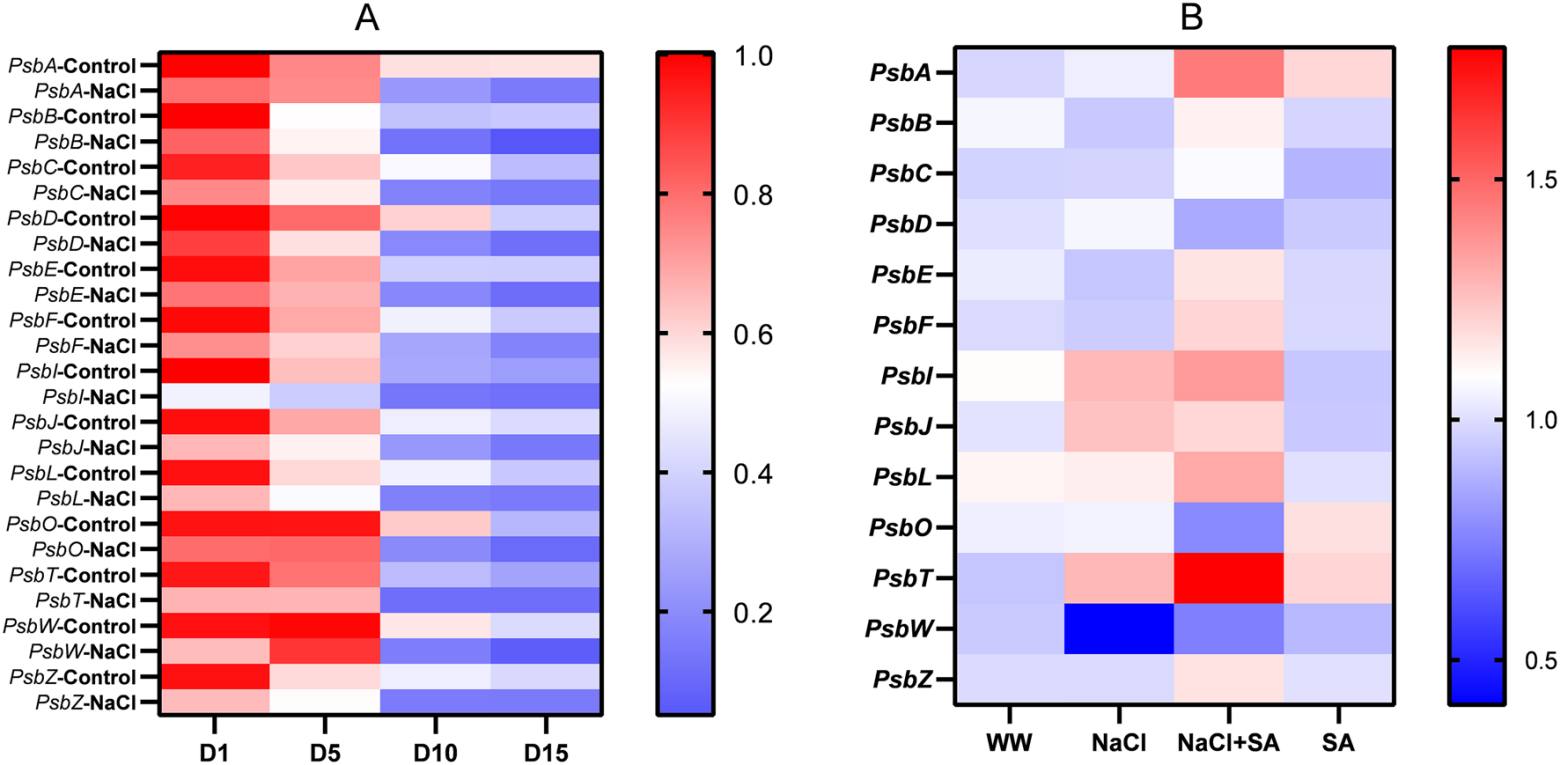
Heatmap of differential gene expression of PSII protein encoding genes. (**A**) Differential Gene Expression of 13 PSII Protein-Encoding Genes in Oat Plants under Normal and Stressed Conditions. Gene expression analyses were conducted at four distinct time intervals (D1, D5, D10, and D15). The color scale represents gene expression levels, with red indicating high expression and blue indicating low expression. (**B**) Gene expression analyses were conducted on 13 PSII protein encoding genes under distinct treatments: WW (well-watered oat plants, control), NaCl (oat plants subjected to incremental salinity stress, 50–400 mM NaCl, for 3 weeks), NaCl + SA (oat plants irrigated with 10 mM sodium acetate (SA) mixed with tap water containing varying NaCl concentrations, 50–400 mM, for 3 weeks), and SA (plants irrigated with 10 mM sodium acetate for 3 weeks). Fold expression values were normalized to the endogenous GAPDH gene expression. Significance (*P* ≤ 0.05) was determined between treatments using one-way ANOVA followed by Tukey's post hoc test (n = 3).

To gain insights into the regulatory role of SA in the molecular mechanism of photosynthetic gene induction, we profiled the expression of 13 PSII-associated genes over 15 days in oat plants exposed to salinity stress (Table 2, Fig. 8A). We observed a gradual reduction in the expression of all 13 genes throughout the experiment. Notably, *PsbI* exhibited a remarkable and significant decrease in expression within just one day of exposure to salt stress. After five days of salt stress, both the *PsbA* and *PsbO* genes had their expression levels halved (Fig. 8A). When compared to control plants, a decrease in the expression was observed in several genes, including *PsbC, PsbD, PsbE, PsbF, PsbI, PsbJ, PsbL, PsbO, PsbT* and *PsbZ* (Fig. 8A). Among these, *PsbI* displayed the most substantial decline in expression. Interestingly, *PsbA* maintained a consistent level of expression in both control and stressed plants. Over the 15-day stressed period, the most significant reduction in gene expression was observed in *PsbA*, *PsbD*, *PsbJ*, *PsbO*, *PsbW* and *PsbZ* when compared to control plants. Interestingly, *PsbW* expression increased after the first day but subsequently experienced a drastic reduction after day 5, persisting until day 15 (Fig. 8A). Taken together, these results show a consistent trend of decreasing expression in all 13 PSII protein-coding genes for 10 days of salt stress, with notable variations among specific genes and time points.

**Table 2:** Identification of PsII-encoding genes and their applications against abiotic stress in plants.

S. No.	Gene	Encoded protein	Role in PS	Role in abiotic stress response	Refs.
1	*PsbA*	D1	Required for oxygen evolution	Protection of PSII from oxidative damage, high light, salinity, drought, and temperature	^38–43^
2	*PsbB*	CP47	Chlorophyll binding inner antenna protein	Response to heat, cold, osmotic stress	^37,44,45^
3	*PsbC*	CP43	Chlorophyll binding inner antenna protein	Responsive to salinity stress	^42,46^
4	*PsbD*	D2	Involved in photodamage repair of PSII	Response to drought and salinity	^42,43,47^
5	*PsbE*	Cyt*b*_559_-α	Involved in PSII assembly, and oxygen evolution	Response to drought and salinity	^48,49^
6	*PsbF*	Cyt*b*_559_-β	Involved in PSII assembly, and oxygen evolution	Unknown	^50^
7	*PsbI*	PsbI	Required for stability PSII-LHCII supercomplex	Unknown	^51^
8	*PsbJ*	PsbJ	Required for proper functioning of OEC, and regulation of electron flow to the plastoquinone pool	Unknown	^52^
9	*PsbL*	PsbL	Required for proper functioning of OEC, and regulation of electron flow to the plastoquinone pool	Unknown	^53^
10	*PsbO*	PsbO	Component of OEC, involved in oxygen evolution and protecting PSII from photo-damage	Responsive to various abiotic stresses	^54–61^
11	*PsbT*	PsbT	Required for efficient repair of photodamaged photosystem II reaction center	Unknown	^62,63^
12	*PsbW*	PsbW	Involved in PSII homodimerization and stability	Unknown	^64,65^
13	*PsbZ*	PsbZ	Required for the interaction between PSII and LHCII complexes	Unknown	^66^

In the subsequent phase, we conducted a comprehensive gene expression analysis for the 13 genes in the four plant groups. Comparing the NaCl plants to the NaCl + SA-treated ones, we observed significantly higher expression levels in *PsbA*, *PsbL*, *PsbT*, and *PsbZ* (Fig. 8B). These findings suggest a potential role of SA application in influencing the expression of PSII proteins encoded by these specific genes. Surprisingly, despite the visibly improved phenotype and enhanced physiology observed in NaCl + SA plants in comparison to NaCl plants, the expression of *PsbO* and *PsbD*, two genes encoding proteins crucial for oxygen evolution during the light-driven reactions of photosynthesis, was lower in the NaCl + SA-treated plants than in the NaCl-treated ones (Fig. 8B). Combined, these results collectively unveil the role of SA in alleviating the detrimental impacts of NaCl on plant photosynthetic parameters. This mitigation is achieved through the molecular regulation of genes encoding crucial proteins within the photosynthetic antenna system.

## Discussion

Exogenous applications of chemical substances have been recognized as an effective and convenient strategy to enhance the tolerance of many crops to various environmental stresses, thereby improving their productivity^21^. Hence, there is a growing focus on exploring novel chemical compounds as signaling molecules to efficiently enhance crop stress tolerance in a cost-effective manner, in comparison to traditional breeding techniques. This trend is evident in an increasing number of studies, as highlighted by Rahman et al.^18^ and Abdelrahman et al.^20^. Despite this, there remains limited knowledge regarding the role of sodium acetate (SA) in mitigating salt stress, particularly concerning its impact on photosynthesis and antioxidant defense mechanisms. To address this gap, our study investigates the potential application of acetate as a chemical agent to enhance physiological and biochemical parameters, alleviating salt-induced toxicity in oat. Our findings demonstrate that exogenous SA supplementation improves salinity tolerance in oat seedlings by preserving photosynthetic pigment levels, enhancing antioxidant defense, and promoting osmoprotection. Initially, we assessed whether SA supplementation could mitigate salt-induced damage in oat plants by examining phenotypic appearance and various growth attributes (Fig. 1). Under salinity stress, seedlings treated with SA exhibited superior morphology compared to those treated with NaCl alone (Fig. 1). This improvement is further evident in the recorded results for growth characteristics, including shoot length, fresh weight, and dry weight of oat seedlings. As depicted in Fig. 1, salt stress negatively impacted shoot length, fresh weight, and dry weight of oat seedlings. However, the application of SA enhanced all these parameters under NaCl stress (Fig. 1). The observed effects of salinity on leaf fresh and dry weights are attributed to a reduction in cell division and cell elongation, consequences of limited CO_2_ assimilation and stomatal closure^19^. Under stress conditions, sodium acetate demonstrated positive effects on morphological aspects. It significantly increased the FW and DW of stressed oat plants almost equivalent to WW-treated plants (Fig. 1). Additionally, it enhanced shoot length in NaCl + SA-treated plants in comparison to those treated solely with SA, even in the presence of salinity stress (Fig. 2) It has been shown in previously studies that Zinc ammonium acetate application led to increased the root volume and weight in maize, promoting a more extensive and active root system that facilitated improved water and nutrient absorption, thereby enhancing overall plant growth^67^. Similarly, in strawberries, acetate treatment contributed to increased shoot growth under stressful conditions by facilitating enhanced water and nutrient supply, consequently boosting FW and DW^68^. Hajihashemi et al.^69^ found that pre-treating quinoa seeds with CaCl_2_, H_2_O_2_, and sodium nitroprusside effectively mitigated the negative impact of salt stress on seed germination. The seeds subjected to pre-treatment exhibited a noteworthy improvement in germination rate, relative germination rate, and germination index. Moreover, the mean germination time was significantly reduced, demonstrating positive effects under both optimal and stress conditions.The capacity for acetate-induced tolerance to abiotic stress has been documented in various plant species, revealing a favorable association with heightened root and shoot biomass, leaf relative water content, and hormonal metabolism and leads to a reduction in oxidative damage^21,22^. Morever, 1 mM acetic acid was found to alleviate salt stress in strawberries, counteracting the salt-induced reduction in leaf and root weight^6^. Hajihashemi et al.^69^ reported that, the application of sodium nitroprusside resulted in the reduction of salt-induced negative impacts on tissue structure and enhanced the levels of photosynthetic pigments, carbohydrates, and proteins, along with increased antioxidant activity. Additionally, it restrained the accumulation of reactive oxygen species (ROS) and lipid peroxidation, ultimately promoting the growth of radish plants experiencing salt stress^69^.

Previously, Kim et al.^70^ documented enhanced drought tolerance induced by acetate, observing a 75% survival rate in Arabidopsis seedlings treated with acetic acid during prolonged drought stress, while the majority of untreated seedlings failed to endure the stress. Additionally, Hossain et al.^71^ demonstrated that acetate-treated seedlings displayed a more robust phenotypic appearance, along with higher shoot and root dry weights compared to their non-acetate-treated counterparts under Cd stress, emphasizing the protective role of this compound in mitigating Cd toxicity in lentil seedlings. The authors suggested that the elevated acetic acid levels in plant cells might function as an energy source, ensuring sufficient cellular energy during challenging conditions^15^.

The degradation of chlorophyll under stress conditions serves as a critical indicator of a plant’s stress tolerance^72^. To further unravel the regulatory impact of SA on oat plant growth, we conducted measurements encompassing chlorophyll and carotenoid content. In our observations, we noted a decrease in chlorophylls and carotenoids in NaCl-treated plants. Conversely, the SA treatment led to an increase in both photosynthetic pigments (Fig. 3). Similarly, there was a significant reduction in Chlorophyll and biomass levels observed in rice, tomato, and wheat plants when exposed to Cd stress conditions^23,73,74^. Conversely, treatments involving acetate preserved leaf chlorophyll content and consequently enhanced the growth and productivity of rice plants facing Cd stress, as indicated by Treesubsuntorn and Thiravetyan^23^.

Further, SA plays a key role in this process by increasing the availability of acetyl CoA, a precursor in the isopentenyl pyrophosphate (IPP) pathway, which in turn enhances carotenoid content^75^. Remarkably, in conjunction with the restoration of chlorophyll content observed in NaCl-treated plants upon the addition of SA (Fig. 3A), we noted a concurrent increase of chlorophyll fluorescence, chlorophyll parameters, phenomenological energy fluxes and the upregulation of genes associated with photosynthetic antenna system (Figs. 7 and 8). Previous studies have linked osmotic stress to reduced maximum photochemical efficiency (Fv/Fm) in various crops like wheat^76^, mango^77^ and rice^78^. It has also been associated with decreased phenomenological energy flux in oat and wheat^37,79^, along with downregulated expression levels of critical genes such as *PsbA*, *PsbB*, *PsbC* and *PsbD* in oat^37^. However, our report is the first to document the reversal of these parameters through SA application under salt-induced osmotic stress conditions. This underscores the ability of SA to sustain the stability of active Photosystem II (PSII) and chlorophyll parameters to a significant extent during salt-induced osmotic stress. Our findings further elucidate the favorable impact of SA application on the number of active reaction centres, trapped electron flux per reaction centre, and electron transport flux per reaction centre (Fig. 7E). Moreover, we provide genetic evidence that SA enhances photosynthetic efficiency by upregulating the expression levels of genes associated with the photosynthetic antenna system, including *PsbA*, *PsbB*, *PsbC*, *PsbE*, *PsbF*, *PsbI*, *PsbJ*, *PsbL*, *PsbT*, and *PsbZ* (Fig. 8B). Table 2 summarizes the role of the genes encoding PSII proteins (intrinsic and extrinsic), analyzed in this study. Intriguingly, our results reveal a novel mechanism whereby SA potentially mitigates stress. Of particular interest is the unexpected decrease in the expression level of *PsbO* mediated by SA application in NaCl-treated plants. This finding contrasts with the predominant higher expression level of this gene observed in drought-tolerant cultivars^61^ and its recognized role in photoprotective functions within PSII^80^. This unexpected contrast raises questions about the intricate relationship between SA application and the regulation of these key genes in the context of photosynthesis, highlighting an area of particular interest for further investigation.

Furthermore, SA demonstrated substantial effects on various other physiological parameters, including electrolyte leakage, antioxidant and proline content (Fig. 4). Accordingly, acetic acid pretreatment also has been shown to enhance cell membrane integrity in common beans subjected to drought stress^81^. Proline plays a multifaceted role in plant stress responses. It serves as a free radical scavenger, regulates osmotic pressure, maintains subcellular structures, and acts as an osmoprotectant to enhance abiotic stress tolerance^58,82^. In our study, oats treated with SA under salt stress conditions exhibited a significant accumulation of proline than plants treated with NaCl. This heightened proline accumulation played a pivotal role in enabling these plants to withstand the challenges posed by salt stress, once proline possesses osmoregulatory properties that help alleviate the detrimental effects of osmotic stress in crop plants^83^. In addition to proline, plants employ a multitude of antioxidant enzymes to counter abiotic stress. These enzymes play a crucial role in scavenging reactive oxygen species from within plant cells, further bolstering the plant's defense against stressors^84^. Antioxidant enzymes protect membranes and DNA from the harmful effects of free radicals generated during abiotic stress. Furthermore, Hossain et al.^19^ demonstrated that the harm inflicted on lentils by salt stress is considerably more pronounced compared to iso-osmotic stress, primarily attributed to a significant decline in AsA content. Shalata and Neumann^85^ proposed that the effective recovery of seedlings from salt stress is associated with the antioxidant activity of AsA, rather than the osmotic aspect of salt stress. Earlier, Hajihashemi et al.^69^ reported that, the application of sodium nitroprusside resulted in the reduction of salt-induced negative impacts on tissue structure and enhanced the levels of photosynthetic pigments, carbohydrates, and proteins, along with increased antioxidant activity. Additionally, it restrained the accumulation of reactive oxygen species (ROS) and lipid peroxidation, ultimately promoting the growth of radish plants experiencing salt stress^69^. To elucidate the functionality of the antioxidant machinery in oat plants during salinity stress with or without the application of sodium acetate, we conducted real-time PCR analysis of the genes encoding these crucial antioxidant enzymes. Our findings revealed that, compared to NaCl-treated plants, NaCl + SA-treated plants exhibited higher gene expression levels for these enzymes. Notably, pre-treatment with acetic acid was previously shown to mitigate salt-induced damage and enhance SOD and CAT levels after three weeks of salt stress in perennial ryegrass^22^. The findings suggest that acetate contributes to ion homeostasis and preserves antioxidant activity in seedlings exposed to salt stress, enabling their recovery from the stress.

The precise mechanisms underlying the beneficial effects of sodium acetate (SA) in mitigating salt stress, particularly concerning hormone accumulation, remain an intriguing area of inquiry. This study seeks to shed light on the potential of sodium acetate to modulate hormone levels in oats subjected to salt stress. The application of exogenous sodium acetate was found to enhance the accumulation of hormones, in the leaves of oat seedlings. Notably, ABA and IAA levels were significantly higher in plants under salt stress compared to control plants (Fig. 6). The accumulation of ABA, in particular, is a well-established response to osmotic stress, as both drought and salt stress elevate the expression of key genes involved in ABA biosynthesis. These genes include *ZEAXANTHIN EPOXIDASE* (*ZEP*), *ALDEHYDE OXIDASE* (*AAO3*), *9-CIS EPOXYCAROTENOID DIOXYGENASE 3* (*NCED3*), and *MOLYBDENUM COFACTOR SULFURASE* (*MCSU*)^86,87^. The role of ABA in stress adaptation is further exemplified by findings in Os*PYL9* mutant lines, which exhibited enhanced ABA accumulation, heightened antioxidant activity, increased chlorophyll content, and improved survival rates under drought stress. Concomitantly, these mutant lines displayed reduced stomatal conductance, diminished malondialdehyde (MDA) content, and decreased transpiration rates^88^. Elevated ABA levels have been observed in various plant species, including *Oryza sativa*^89^, brassica^90^, *Phaseolus vulgaris*^91^, and *Zea mays*^92^. As ABA levels rise, proteins accumulate for osmotic adjustment, closing the stomata. Furthermore, auxin accumulation and growth rate were positively correlated, indicating that auxin plays an important role in photomorphogenesis. According to a recent report^93^, over-expression lines exhibit increased auxin accumulation, improved lateral root development, and enhanced enzymatic antioxidant defenses (CAT, APX, SOD, and GPX).

Upon conducting a thorough analysis of the presented data, a slight adverse effect of applying SA on plant growth is evident under non-saline (control) conditions. However, it is important to note that the difference in growth parameters between SA and WW treatments is not statistically significant, as observed in the data collected after three weeks of stress treatment. This trend is reflected in Fig. 1, captured after the fifth week of treatment. While the reduction in the growth of SA-treated plants under non-saline conditions compared to WW plants is not highly significant, it may be attributed to the gradual accumulation of SA in both potting soil and plants as the treatment progresses. The observed minor negative impact of SA application on plant growth emphasizes the necessity for an improved methodology in its application, rather than the continuous inclusion of SA in irrigated water. Hence, a more focused research is required to optimize the positive impact of SA application in mitigating the negative effects of salt stress, considering factors such as dosage, frequency, and mode of application, etc.

## Conclusion and future prospective

**Figure 9:**
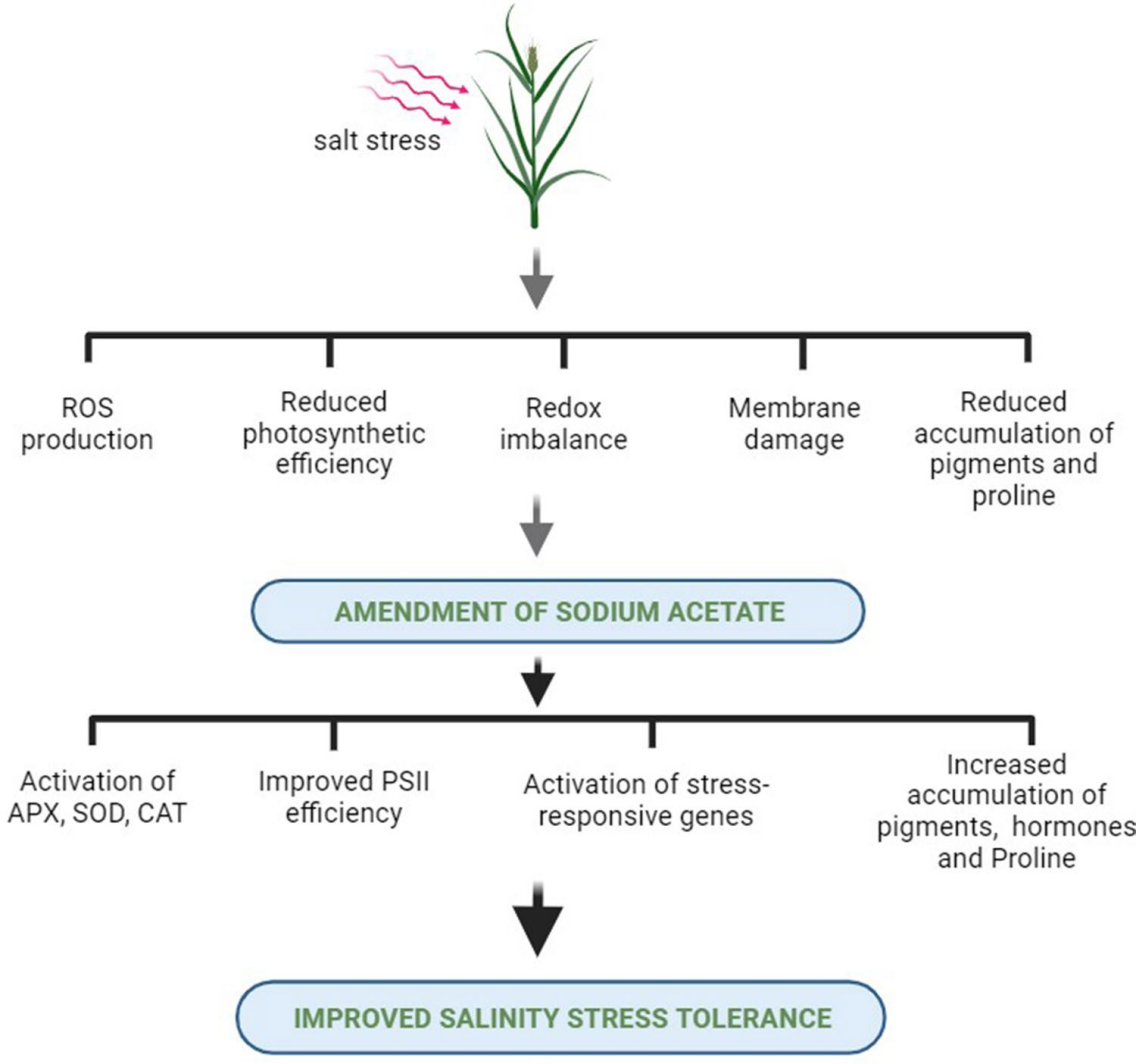
A Hypothetical Model Illustrating the Effects of Sodium Acetate Application for Inducing Salinity Stress Tolerance in Oat Plants. This proposed mechanism involves elevating proline levels and enhancing the antioxidant enzyme system, consequently leading to improved PSII efficiency and increased accumulation of plant pigments. Diagram created using BioRender.com.

Our findings unveil the activation of a protective mechanism induced by SA under salt stress conditions (Fig. 9). This mechanism is characterized by an increase in proline levels and the bolstering of the antioxidant enzyme system. These alterations appear to contribute, at least in part, to the upregulation of gene expression related to photosynthesis, the enhancement of photosynthetic parameters, and the elevated levels of chlorophyll and carotenoid content observed in our study (Fig. 9). Moreover, the results presented here identify the pivotal role of SA as a critical factor that can be harnessed to improve the productivity of oat plants in the context of salinity stress. These acetate-induced enhancements effectively mitigated oxidative damage caused by salinity, preserved the integrity of plant cell membranes, and subsequently improved the tolerance of oat plants to salt stress. Our findings suggest that acetate could serve as a practical and cost-effective solution for farmers to manage salt stress in crop plants. Additional research is needed to uncover the molecular mechanisms underlying the acetate-induced enhancement of salt tolerance in plants as well as to develop an efficient methodology for its application in agriculture for improved growth and yield of plants grown under saline evironments.

### Supplementary Information

Supplementary Information.

## Data Availability

All data generated or analysed during this study are included in this published article and its supplementary information files.
