# Supplementary material for: Modulation of antioxidant defense and PSII components by exogenously applied acetate mitigates salinity stress in Avena sativa
Source: Sci Rep. 2024 Jan 5;14:620. doi: 10.1038/s41598-024-51302-5 (PMC10770181; doi:10.1038/s41598-024-51302-5)
Supplement: Supplementary file 1 — Supplementary Information. [file 41598_2024_51302_MOESM1_ESM.docx]

**Table S1:** Primers sequences used in qPCR for amplifying 17 *Avena sativa* genes.

| **No.** | **Primer name** | **Sequence 5’ to 3’** |
| --- | --- | --- |
| 1 | As-*PsbA*-F | TCGCTTCTGCAACTGGATAAC |
|  | As-*PsbA*-R | GCAGCGATGAAGGCGATAATA |
| 2 | As-*PsbB*-F | GCGGGTACGTTGGGTATATTAG |
|  | As-*PsbB*-R | CAGCAGCGATACTACTGGAAAG |
| 3 | As-*PsbC*-F | AACGGTTTGGACTTGAGTAGG |
|  | As-*PsbC*-R | CTACGCCACCCACAGAATTTA |
| 4 | As-*PsbD*-F | GCCCTTGGTAGAGTTCCTAAAG |
|  | As-*PsbD*-R | CGAAATAAGCGCAAGGAAAGAG |
| 5 | As-*PsbE*-F | TCCCTATTCATTGCGGGTTG |
|  | As-*PsbE*-R | TGTCGGCTTTCCGTGAAATA |
| 6 | As-*PsbF*-F | TGCGATGGCTGGCTATTC |
|  | As-*PsbF*-R | TTATCGTTGGATGAACTGCATTG |
| 7 | As-*PsbI*-F | ACAGTAGTGATATTCTTTGTTTCCC |
|  | As-*PsbI*-R | CAGGATTCCGTCCTGGGTC |
| 8 | As-*PsbJ*-F | CCTCTTTGGCTGATAGGTACTG |
|  | As-*PsbJ*-R | AGATGAACCCAATCCAGAATATGA |
| 9 | As-*PsbL*-F | TGACACAATCAAACCCGAATGA |
|  | As-*PsbL*-R | ACAAAAATGAGTAATAAACCCCAGT |
| 10 | As-*PsbO*-F | AGAGAGGCTCGGTGAAATAGA |
|  | As-*PsbO*-R | CCAATCCCAGGGAACAGTAAAG |
| 11 | As-*PsbT*-F | GGAAGCATTGGTTTATACGTTCC |
|  | As-*PsbT*-R | GTGGAACCTTAGGTGGTTCTC |
| 12 | As-*PsbW*-F | CTCGGGCTGAGCAACAA |
|  | As-*PsbW*-R | TCGGAGGTGTAGATGGTGTA |
| 13 | As-*PsbZ*-F | GACTATTGCTTTCCAATTAGCTGT |
|  | As-*PsbZ*-R | TTGACCAACCATCAGGAGAAG |
| 14 | As*GAPDH*-F | GGTGGTGCCAAGAAGGTTAT |
|  | As*GAPDH*-R | GGAGACAATGGTGATGTCAGAG |
| 15 | As- *CAT1*-F | CAGGCTGGCGAGAGATTCC |
|  | As- *CAT1*-R | AGCATCCGTGAGTGCATCAA |
| 16 | As- *APX*-F | GCTCCGTGAAGTAAGTGTTATCAAAC |
|  | As- *APX*-R | CCTGGGAAGGTGCCACAA |
| 17 | As- *SOD*-F | CACAAGCACTTCACAGGAACAGT |
|  | As-*SOD*-R | TGCCACTCTGAACATTTCATCAC |

Primers 1-14 were designed based on cDNA sequences retrieved from a transcriptome assembly of *Avena sativa* generated (unpublished) within our lab. Sequence of primers 15-17 were taken from a previously published article by Kong et al., 2015.

1. Kong, L., Huo, H. & Mao, P. Antioxidant response and related gene expression in aged oat seed. *Front Plant Sci* **6,**158. (2015).
